# A deterministic model for the occurrence and dynamics of multiple mutations in hierarchically organized tissues

**DOI:** 10.1098/rsif.2013.0349

**Published:** 2013-08-06

**Authors:** Benjamin Werner, David Dingli, Arne Traulsen

**Affiliations:** 1 Evolutionary Theory Group, Max Planck Institute for Evolutionary Biology, Plön, Germany; 2 Division of Hematology, Mayo Clinic, College of Medicine, Rochester, MN, USA

**Keywords:** cancer, clonal extinction and diversity, mathematical modelling

## Abstract

Cancers are rarely caused by single mutations, but often develop as a result of the combined effects of multiple mutations. For most cells, the number of possible cell divisions is limited because of various biological constraints, such as progressive telomere shortening, cell senescence cascades or a hierarchically organized tissue structure. Thus, the risk of accumulating cells carrying multiple mutations is low. Nonetheless, many diseases are based on the accumulation of such multiple mutations. We model a general, hierarchically organized tissue by a multi-compartment approach, allowing any number of mutations within a cell. We derive closed solutions for the deterministic clonal dynamics and the reproductive capacity of single clones. Our results hold for the average dynamics in a hierarchical tissue characterized by an arbitrary combination of proliferation parameters. We show that hierarchically organized tissues strongly suppress cells carrying multiple mutations and derive closed solutions for the expected size and diversity of clonal populations founded by a single mutant within the hierarchy. We discuss the example of childhood acute lymphoblastic leukaemia in detail and find good agreement between our predicted results and recently observed clonal diversities in patients. This result can contribute to the explanation of very diverse mutation profiles observed by whole genome sequencing of many different cancers.

## 1. Introduction

The lifespan of most cells in biological organisms is limited, and usually the life expectancy of the organism exceeds this time by orders of magnitude [1,2]. As cells are continuously lost, mechanisms to replenish the cell pool have evolved in organisms, enabling sustained cell production during the lifetime [3]. Often this is realized by hierarchically organized tissue structures. At the root of the hierarchy are a few tissue-specific stem cells, combining two properties—self-renewal and differentiation potential [4]. During cell proliferation, cells differentiate and become increasingly specialized to perform specific functions within the hierarchy. After some differentiation steps, the complete spectrum of functional cells can be obtained [5–10]. A prominent example is the haematopoietic system [6–11], but other tissues such as skin [12,13] or colon [14] are also hierarchically organized.

A large number of cell divisions are indispensable to life; however, they are unavoidably accompanied by mutations. Typically, these cells are washed out of the hierarchy and thus, especially if they arise in relatively differentiated cells, the associated mutations are lost in the long run [10,15]. But cells with multiple mutational hits might persist for a long time, increasing the risk of accumulation of additional mutations during cell proliferation, which can ultimately lead to cancer. Although some cancers seem to be caused by single mutation hits, for example the BCR-ABL oncogene occurring in stem cells in chronic myeloid leukaemia [16] or the PML-RARA oncogene occurring in more differentiated cells in acute promyelocytic leukaemia [17], they are rare. The majority of cancers are triggered by at least a handful of mutations [18–20]. The recent progress in genome sequencing techniques has allowed, in some cases, the classification of cancer-initiating mutations; in other cases, the underlying mutations remain unknown [21]. However, many of these studies reveal a very diverse mutation landscape, indicating the existence of several cancer-initiating driver mutations and additional alterations that have a small or even no impact on cancer development, the so-called passenger mutations [22–29]. The precise impact of passenger mutations on cancer progression is still under discussion, but the typical assumption is that they are neutral and do not affect the proliferation properties of cells [30,31]. In particular, this holds for synonymous mutations that do not have any consequences for protein structure or function [32].

In mathematical and computational approaches, compartment models are frequently used to describe cell dynamics in hierarchically organized tissue structures. Many of these studies investigate the effects of stem cell mutations and the related clinical implications [6,8,33–36]. Also the stochastic effects of tissue homeostasis are analysed [37–39], highlighting that cancer-driving mutations can in principle disappear by chance (stochastic extinction). The interplay of stem cell and progenitor cell mutations and their impact on cancer initiation are discussed [40], and game theoretical approaches allow modelling of the evolutionary aspects of tissue homeostasis and intercell competition [41,42]. Often, these studies investigate the effects of cells carrying one or a very few specific mutations and assume either constant population size or only minimal hierarchies.

Here, we focus on the presence of cells carrying multiple mutations within a hierarchically organized tissue. We show mathematically that the hierarchical organization strongly suppresses cells carrying multiple mutations and thus reduces the risk of cancer initiation. Closed solutions for the total cell population that arises from a single (mutant) cell are derived, and from this the expected diversity of the mutation landscape and the clonal size can be described. This enables a better understanding of the expected diversity in mutation landscapes that are observed in both healthy and cancerous tissues.

### 1.1. Mathematical model

**Figure 1.**
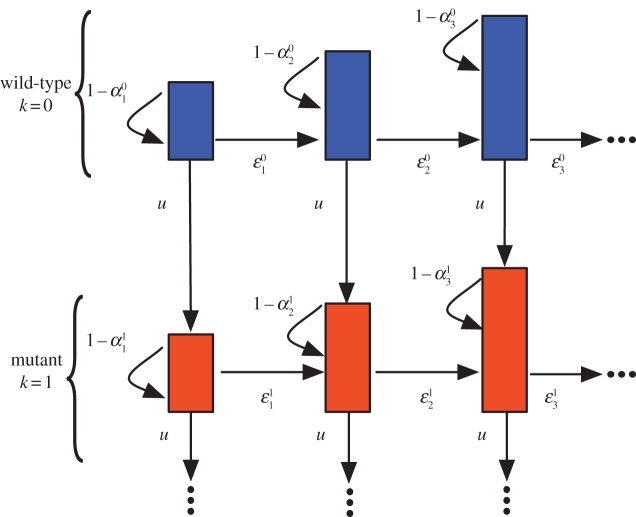
Schematic of the compartment structure of multiple mutations and the corresponding transition rates. The top compartments contain cells carrying no mutation. The bottom compartments contain cells carrying one mutation. Compartments to the right represent more specialized cell stages and arrows represent transition probabilities, where *ɛ* denotes the differentiation probability, *u* denotes the mutation rate of cells and 
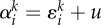
. Initially, no mutated cells are present in the hierarchy. We then determine, how many cells are acquired from the founder compartment (top left) and investigate how many cells with *k* mutations are on average expected at any stage of the hierarchy. (Online version in colour.)

The hierarchical tissue organization is typically modelled by a multi-compartment approach [6,10]. Each compartment represents a certain differentiation in the stage of cells. At the root of the hierarchy are stem cells ensuring a continuous influx of cells. A proliferating cell in compartment *i* divides and the two daughter cells differentiate and migrate into the next downstream compartment (*i* + 1) with probability *ɛ*, increasing the downstream compartment by two cells, mutates with probability *u* or self-renews within its own compartment with probability 1 − *ɛ* − *u*. Mutated cells stay in the hierarchy. If a mutated cell proliferates, it differentiates with probability *ɛ* into the next downstream compartment, it self-renews with probability 1 − *ɛ* − *u*, or it mutates with probability *u* again, leading to a cell with two (or more) mutations. All possible outcomes of a cell proliferation are depicted in figure 1. The directions of the arrows point towards the accessible cell states and the labels give the transition probabilities. We allow arbitrary parameters and introduce 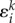 as the differentiation probability of cells in compartment *i* carrying *k* mutations. Asymmetric cell divisions are not explicitly implemented, as they can be absorbed in the differentiation probabilities on the population level. The fate of a cell's offspring is determined based on the probabilities 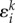. Cells proliferate with a rate *r_i_* in each compartment *i*. Usually, cells in upstream compartments proliferate slowly and cell proliferation speeds up in downstream compartments (i.e. *r_i_* < *r_i_*_+__1_). This general framework is very flexible and different tissue structures can be represented.

### 1.2. Stochastic individual-based simulations

We implement individual-based stochastic simulations of the cell dynamics in hierarchically organized tissue structures. We use an implementation of the Gillespie algorithm [43,44]. Originally introduced to simulate chemical reactions, it allows us to reproduce exact stochastic trajectories of the system. Each cell has an individual representation. Thus, the complete clonal history of cells within the hierarchy can be recorded. If a cell is chosen for reproduction (determined by the Gillespie method), it differentiates, self-renews or mutates according to the probabilities 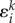 and *u*. The parameters of the simulated system are described later and chosen to represent human haematopoiesis.

### 1.3. The haematopoietic system

In the following, we focus on the haematopoietic system. There, approximately 400 stem cells replenish the haematopoietic cell pool [45,46]. Each stem cell divides approximately once a year [45,47]. Cell proliferation is assumed to increase exponentially with compartment number *i*, *r_i_* = *γ*^*i*^*r*_0_, with *γ* = 1.26 and *r*_0_ corresponds to the proliferation rate of stem cells. The differentiation probability is assumed to be constant, *ɛ* = 0.85, for all non-stem cell compartments, and in total *i* = 31 compartments are needed to ensure a daily bone marrow output of approximately 3.5 × 10^11^ cells [6,10].

## 2. Results

### 2.1. Time continuous dynamics of multiple mutations

**Figure 2.**
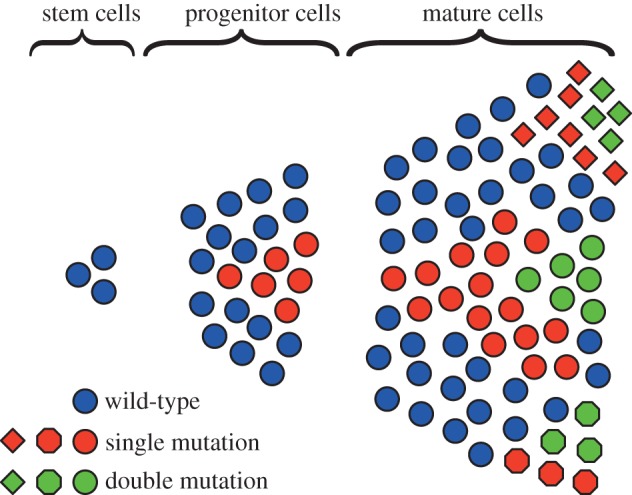
Clonal expansion within a hierarchically organized tissue. Cell proliferation is driven by a few slow-dividing stem cells, giving rise to faster dividing progenitor cells. After some differentiation steps, the mature tissue cells are obtained. Initially cells have no mutations, but mutants can arise and expand within the hierarchy. These cells either vanish or gain an additional mutation, which again potentially spreads within the hierarchy. Different colours code for a different number of mutations, whereas different shapes indicate different mutations. (Online version in colour.)

We describe the deterministic dynamics of a cell population within a hierarchically organized tissue structure, which initially carries no mutation. A cell may commit further into the hierarchy (differentiate), mutate or self-renew. This occurs with probability *ɛ*, *u* and 1 − *ɛ* − *u*, respectively. In figure 1, a schematic of the resulting hierarchical structure is shown. Compartments to the right represent downstream compartments of more specialized (differentiated) cells, while compartments to the bottom represent states of cells which accumulated an additional mutation. During one cell division, a cell either mutates and moves one compartment to the bottom, differentiates and produces two cells in the next downstream compartment to the right or self-renews and produces an additional cell within its original compartment. This leads to an expansion of clonal populations within the hierarchy that potentially accumulates several (distinct) mutations during the differentiation process. This is schematically shown in figure 2.

The above transition probabilities can be used in an individual-based stochastic simulation. In the following, we provide a deterministic description of the average dynamics of cells carrying multiple mutations in such hierarchical structures. Thus, we describe the dynamics by transition rates instead of transition probabilities, but only averages are included in our description. By doing so, we neglect certain effects, such as stochastic extinction of cells. However, the approach allows us to investigate the averages of the underlying stochastic simulations.

Assume that in compartment *i* there are 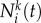 cells carrying *k* mutations at time *t*. The number of these cells increases as a result of influx from the upstream compartment at a rate 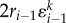, mutations at a rate *r_i_u* and self-renewal at a rate 
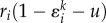
. Cells are lost either by mutation at a rate *r_i_u* or by differentiation at a rate 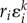. The deterministic description of the hierarchical compartment model becomes a system of coupled differential equations [10], given by2.1
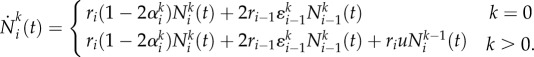
Here, 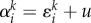 denotes the probability that a cell with *k* mutations leaves compartment *i*. Typically, 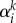 is very close to *ɛ*. A model for stochastic cell dynamics in the stem cell compartment for neutral and non-neutral mutations can be found in [39,48]. In those papers, the stochastic Moran process is used to investigate the extinction and fixation probabilities of stem cell mutations. The deterministic stem cell-driven cell replenishment in hierarchical tissues is studied in detail in [10]. However, in that prior work, the effects arising from additional mutations were neglected. Here, we focus on non-stem cell-driven clonal dynamics. We explicitly allow for an arbitrary number of mutational hits at any stage of the hierarchy, but we neglect a continuous influx of mutated cells from the stem cell level. This assumption gives the condition 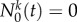. The initial condition2.2
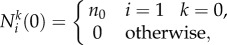
corresponds to initially *n*_0_ cells in compartment 1 carrying no mutation. One can imagine a neutral marker approach, in which one cell in the hierarchy is genetically marked, and one considers the clonal population arising from this marked cell [49]. Although we neglect a continuous influx of mutated cells from the stem cell compartment, stem cell mutations can be implemented indirectly. Our approach allows for altered cell proliferation properties of the founder cell, potentially derived by a mutation at the stem cell level. For a constant differentiation probability, i.e. the case where all 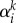 and all 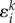 are identical, equation (2.1) can be solved recursively. The number of cells in compartment *i* carrying no mutation changes with time as2.3
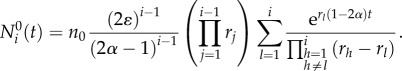
The solution can also be derived recursively for cells carrying *k* mutations in compartment *i* and becomes2.4
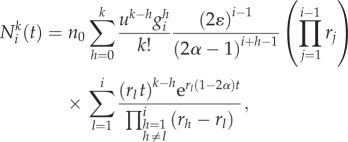
where 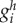 is a combinatoric parameter which is given for cells carrying up to three mutations by2.5
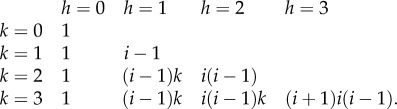
If *α* < 0.5, non-stem cells will continuously accumulate in downstream compartments. The probability of self-renewal in this case is larger than the probability of differentiation. This scenario seems to be realized in certain blood cancers. For example, in acute promyelocytic leukaemia an abnormal increase in immature granulocytes and promyelocytes is observed, resulting from a block of cell differentiation at a late progenitor cell stage [17,50]. However, these cases are rare.

**Figure 3.**
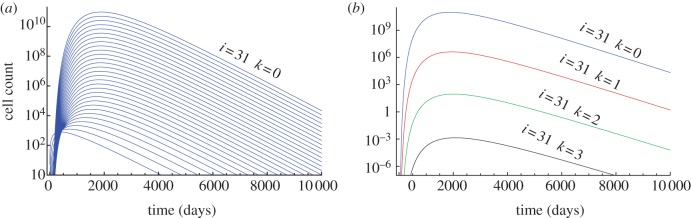
(*a*) Number of cells carrying no mutation in compartments 1–31 arising from compartment 1 containing 1000 cells. Lines show equation (2.3), with parameters *n*_0_ = 1000, *ε* = 0.85, *γ* = 1.26, *u* = 10*^−^*^6^ and *r*_0_ = 1/400. Cells are more likely to differentiate than to self-renew and thus progressively travel into more committed compartments. Initially, the cell count increases, but cells get washed out in the long run. The time scale is determined by the number of stem cell divisions. A stem cell is assumed to divide once a year, thus after 400 stem cell divisions a year has passed. (*b*) Count of cells carrying 0–3 mutations in compartment 31, given by equation (2.4). We used the same parameters as in (*a*). Cells carrying multiple mutations are exponentially suppressed. (Online version in colour.)

For *α* > 0.5, the solution becomes a clonal wave, travelling through the hierarchy in time. In this case, the probability of differentiation is larger than the probability of self-renewal and thus cells progressively travel downstream (figure 3). The cell population founded by a single non-stem cell expands within the hierarchy initially, but gets washed out and vanishes in the long run. This is believed to be true for healthy homeostasis. For example, for the haematopoietic system, the differentiation probability was estimated to be *ɛ* = 0.85 [6]. As by far the most cell proliferations occur at the progenitor and more committed differentiation stages, this provides a natural protection for the organism against the accumulation of multiple mutations, as the survival time of most (non-stem cell-like) mutations is finite.

The maximum mutant cell count of the clonal wave and the time to reach this maximum can be calculated for the compartment of the mutant origin, in our case the first compartment. The time is given by2.6
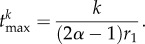
The time to reach the maximum increases linearly with the number of additional mutations *k*. The cell count at the maximum becomes2.7
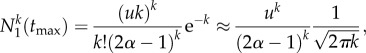
where we used the Stirling formula to approximate *k*!. The maximum scales with *u^k^* and thus decreases exponentially with *k*. In addition, the factor 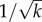 leads to a further suppression of the maximum for increasing *k*. However, the risk of additional mutations depends not only on the maximal cell count but also on the reproductive capacity of a cell line. This reproductive capacity can be captured by the cumulative cell count. The number of cells within compartment *i* carrying *k* mutations produced until time *t* is given by2.8
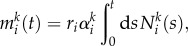
and the reproductive capacity can be derived by taking the time limit to infinity. The general solution (2.4) allows us to carry out the integral exactly by integration by parts. However, the problem can be tackled from a different perspective, leading to a more transparent solution of (2.8) that is easier to handle.

### 2.2. Cell reproductive capacity

We call the cell subpopulation within a compartment *i*, which is derived by a single founder cell in an upstream compartment, the reproductive capacity of this founder cell. This idea directly corresponds to the method of neutral markers. We imagine a genetically marked cell somewhere in the hierarchy and count the offspring of this cell at any stage of the hierarchy. This corresponds to the total count of cells with the same colour in figure 2.

Assume a single cell carrying no mutation in compartment 1. This cell differentiates with probability 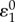 into the next downstream compartment, mutates with probability *u* or produces an additional cell in compartment 1 with probability 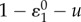. We first discuss the probability that a cell leaves compartment 1 after exactly *l* cell divisions. A cell can leave a compartment either by mutation or by differentiation, before which the cell has to undergo *l* − 1 self-renewals. Thus, this probability becomes (*ɛ* + *u*)(1 − *ɛ* − *u*)*^l^*^−1^. During this time, the cell population in compartment 1 derived from this single cell increases to 2*^l −^*^1^ cells, if all daughter cells share the same proliferation probabilities. With this, the reproductive capacity of a single cell in compartment 1 is on average2.9
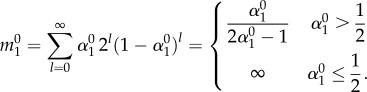
The sum becomes infinite if *α* ≤ 0.5, as the probability of producing offspring in the founder compartment is higher than the probability of leaving the compartment. Thus, the cell population continuously increases. Of course, under normal conditions, cells do not have an unlimited capacity to divide and serial telomere erosion, among others, will impose a physical limit on the number of divisions a cell can undergo [1,51]. The biologically more relevant case is *α* > 0.5 and cells tend to differentiate into more committed compartments. In this case, the total number of offspring cells that arise from a single cell (i.e. a clone) is finite and given by (2.9). The number of cells 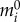 in compartment *i* carrying no mutation increases because of the influx of cells via differentiation from compartment *i* − 1 and the expansion of these cells owing to self-renewal in compartment *i*. Thus, we can write for the reproductive capacity of cells in compartment *i* without mutation2.10
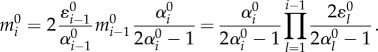
This can be generalized, and an expression for the reproductive capacity of cells in compartment *i* carrying *k* mutations can be derived. Cells in compartment *i* with *k* mutations are acquired by differentiation of cells from compartment *i* − 1 that carry *k* mutations, by mutation of cells in compartment *i* carrying *k* − 1 mutations or by self-renewal of cells already in compartment *i* and *k* mutations. With this, we can write2.11
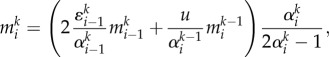
where the two terms in the brackets represent cells produced either by differentiation or by mutation, multiplied by the self-renewal potential of these cells. This recurrence relation can be solved recursively2.12
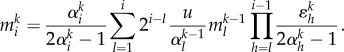
As 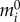 is given by (2.10), one can construct the explicit solution iteratively. Equation (2.12) allows for arbitrary parameters 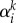 and thus incorporates any mutation-induced change in the cell proliferation parameters. However, if 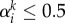 the sum diverges. Cells with at least *k* mutations will accumulate in all compartments downstream of *i*. Note that equation (2.12) captures the general deterministic dynamics of a cell lineage founded by a single cell somewhere in the hierarchy. We are especially interested in the case where this founder cell carries critical, potentially cancer-driving mutations that will allow us to address the expected number of mutations arising in this mutant clone. In addition, the probability of obtaining such a critical mutational hit can be investigated. But even if a cell accumulated a critical number of mutations, it might still become extinct because of stochastic effects [19,37,39]. In the following, we discuss the general solution of equation (2.12) for mutations that are neutral relative to the founder cell.

### 2.3. Reproductive capacity of neutral mutants

**Figure 4.**
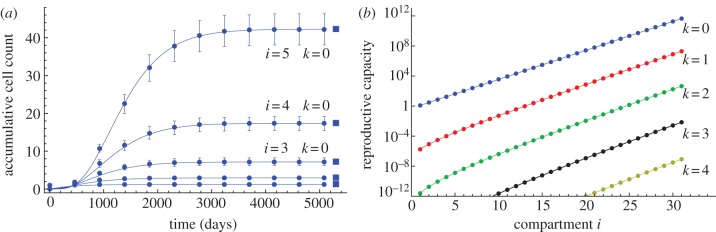
(*a*) Number of cells without mutations in compartments 1–5, arising from a single cell in compartment 1. Lines are equation (2.8), symbols are averages with corresponding standard deviations over 10^3^ independent runs of stochastic individual-based computer simulations, and squares are equation (2.13). Parameters are *n*_0_ = 1, *ɛ* = 0.85, *γ* = 1.26, *u* = 10*^−^*^6^ and *r*_0_ = 1/400. (*b*) Reproductive capacity of a single founder cell in compartment 1. Shown is the number of cells with 0–4 mutations in the first 31 compartments, acquired from a single cell in compartment 1. Symbols are numerical solutions of (2.8) in the limit of infinite time and lines are equation (2.13). The reproductive capacity increases exponentially for increasing compartment number. Cells carrying multiple mutations are strongly suppressed within a hierarchical tissue structure (see equation (2.14)). (Online version in colour.)

We call a mutation neutral if the reproductive capacity of the mutant and the founder cell is equal. In §2.2, we have shown that the reproductive capacity of a cell depends on its differentiation probability *ɛ* and its mutation rate *u*, but interestingly it is independent of the reproduction rate *r*. Therefore, the clonal lineage and the number and type of mutations that arise from a single founder cell do not depend on the proliferation rates of the founder cell. However, the time to reach those states of course depends on *r*. Therefore, although two mutations lead to the same outcome, this might occur on distinct time scales, with observable differences in the progression of diseases. Nonetheless, our definition of neutral mutations only requires constant differentiation probabilities and mutation rates relative to the founder cell. This assumption allows us to write 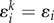 and thus the number of parameters is reduced from (*k* + 1) *i* + 1 for the general case to *i* + 1 for the neutral case. This number can be reduced to two parameters, *u* and *ɛ*, if a constant differentiation probability for all non-stem cell stages is assumed, *ɛ*_*i*_ = *ɛ*. This simplifies the evaluation of the recurrence relation (2.12) significantly. The reproductive capacity 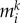 of neutral mutations in compartment *i* carrying *k* mutations becomes2.13
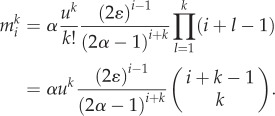
Mutants carrying *k* mutations are suppressed by a factor *u^k^* and thus are rare in the early differentiation stages. The number increases exponentially for downstream compartments, and a significant load of cells carrying few mutations can be observed in the late differentiation stages (figure 4).

Equation (2.13) reveals interesting properties of hierarchical tissue structures. The ratio of cells carrying *k* mutations to cells carrying *k* − 1 mutations in compartment *i* is2.14
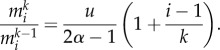
The ratio increases with compartment number, but the increase becomes flatter for increasing *k*. The compartment structure leads to an additional suppression of cells carrying multiple mutations and thus is a protection mechanism against cancer initiation. The ratio is constant for *i* = 1, the compartment of the mutant origin. The protection mechanism affects only downstream compartments.

On the other hand, the scaling properties for more differentiated cells also show interesting properties. The ratio of cells with *k* mutations in compartment *i* + 1 to cells with *k* mutations in compartment *i* is given by2.15
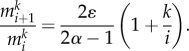
The increase in cells is constant for cells carrying no mutations, *k* = 0. It increases with *k*, but is suppressed within the hierarchy by a factor of 1/*i*.

### 2.4. Number of distinct neutral mutations

So far, we have discussed the reproductive capacity of cells. However, we did not distinguish between different mutations, but grouped together cells with an equal number of mutations. Often, experimental studies focus on mutation landscapes, investigating the variation in clonal loads in healthy and sick individuals. Our approach allows us to estimate the expected number of distinct mutations that arise from a single founder cell, corresponding to the count of distinct symbols with the same colour in figure 2.

Let us assume that every mutation event is unique. Thus, we neglect the possibility that the same mutation is derived twice independently. The diversity in compartment *i* increases either by additional mutations of cells in compartment *i* or by differentiation of clones from compartment *i* − 1 into compartment *i*. Assuming a differentiation probability *ɛ* > 0 the expected diversity 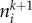 of cells with *k* + 1 mutations in compartment *i* is2.16
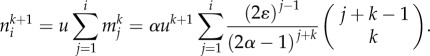


As an example, if we use the parametrization of the haematopoietic system, *ɛ* = 0.85 and *u* = 10*^−^*^6^, we find approximately 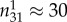 distinct single mutations in compartment 31 that were derived from the clonal progeny of a single founder cell in compartment 1. But the founder cell by chance could carry a mutation that changes their cell proliferation parameters. For example, the differentiation probability of the founder cell could change to *ɛ* = 0.75. In this case, equation (2.16) estimates 28 000 distinct mutations derived from this single founder cell. We note that the above change in the differentiation probability from 0.85 to 0.75 is sufficient to explain the manifestation of chronic myeloid leukaemia in otherwise healthy adults [38,52]. However, equation (2.16) represents an average, and in individual cases fluctuations, caused by stochasticity, are expected. But also small changes in *ɛ* or in *u* influence the expected diversity significantly. A linear error analysis [53] reveals the dependency of (2.16) on the uncertainties of *u* and *ɛ*, which is given by2.17
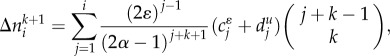
with 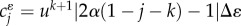 and 
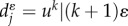
 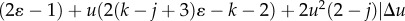. If we assume that *Δu* = 10*^−^*^7^ and *Δɛ* = 0.01 the uncertainty given by (2.17) becomes 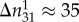, where the individual error contributions in *u* and *ɛ* are 7 and 28, respectively. Especially, note the strong dependency on *Δɛ*. If we choose *Δɛ* = 0.05, a deviation that might be difficult to detect *in vivo*, one gets 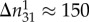. Thus, small variations in *ɛ* lead to significant differences in the expected diversity of clonal populations, one aspect that might contribute to the explanation of the observation of very diverse mutation landscapes. Note also that an increasing mutation rate *Δu* = 10*^−^*^6^ gives 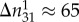. Of course, higher mutation rates increase the expected diversity of clonal populations. However, a higher mutation rate (or genomic instability) is neither the exclusive nor necessarily the dominant underlying cause of the diversity in the mutation landscape that is observed.

### 2.5. Example: clonal diversity in acute lymphoblastic leukaemia

Let us now consider a specific example using data from childhood acute lymphoblastic leukaemia (ALL). The most common chromosomal abnormality in this disease is the t(12;21) translocation that results in the fusion gene ETV6–RUNX1 (also known as TEL/AML1). There is evidence that this mutation often arises *in utero*. This has been confirmed to be the case in at least one pair of monozygotic twins [54]. This mutation is a founder mutation and is considered to be critical for the disease. Cells that express this fusion gene appear to have a higher self-renewal rate and enhanced survival when compared with normal cells [54,55]. In our model, enhanced self-renewal implies a reduced differentiation probability for the cells carrying the mutation (*ɛ* < 0.85). Recently, Ma *et al*. [56] performed whole genome sequencing on leukaemic cells isolated from two pairs of monozygotic twins. In one pair of twins, the initial event occurred *in utero* since the ETV6–RUNX1 fusion was shared by both siblings. They found that the incidence of non-synonymous single nucleotide mutations between the samples ranged from 708 to 1237 [56]. These mutations must have occurred after the founder mutation and independent of each other. The time from the putative appearance of the shared ETV6–RUNX1 mutation and disease was 48–55 months. The second pair of monozygotic twins shared a mutation in NF-1, and the time to diagnosis of ALL was 72–77 months after birth. The tumours in these two children had 949–975 unique non-synonymous single nucleotide mutations. Using these constraints and an estimate of 10–75% cancer cells at diagnosis, unchanged proliferation rates as well as *N*_0_ = 100 [57], we use equation (2.4) to estimate the differentiation probability *ɛ* for the mutant cells, which is then in the range of 0.78–0.81, i.e. only slightly lower than that of normal cells. Based on equation (2.4), it takes approximately 50–80 months to reach this load. We further predict a range of 350–2600 distinct mutations by using equation (2.16), assuming a mutation rate of *u* = 10*^−^*^6^ and the above range of differentiation probabilities. Thus, we expect slightly more distinct mutations than found in patients. But some of those theoretically predicted clonal populations are small and may escape detection. For example, if we neglect mutations that occur during the last differentiation step of cells in compartments 30–31 (expected to be small cell populations), we predict only 150–950 distinct mutations. Clearly, the model as presented can explain the large number of passenger mutations that can be expected in a typical patient with ALL and probably other types of leukaemia.

## 3. Discussion

The accumulation of multiple mutations in cells is considered to be critical for cancer initiation. However, mutations unavoidably accompany cell proliferation and thus cancer can potentially occur in any multicellular organism. Hierarchical tissue structures contribute to the protection against such mutations. So far, the suppression of single mutations in hierarchical tissue structures has been the focus. Here, we have shown that, in addition, the risk of the accumulation of multiple mutations is dramatically reduced by a hierarchical tissue organization. The cell population is divided into a few slowly proliferating stem cells and many faster proliferating progenitor cells. Stem cells have an almost infinite cell reproductive capacity, but the manifestation of a critical mutational load often exceeds an organism's expected natural lifetime. Progenitor cells proliferate faster, but their reproductive capacity usually is limited, and thus they give rise to clonal waves travelling through the hierarchy. Still, both cases can be observed. There are cancers that presumably originate from stem cell mutations, for example chronic myeloid leukaemia, and there are cancers that originate in the later stages of haematopoiesis, for example acute promyelocytic leukaemia and various other subtypes of acute myeloid leukaemia [17]. Understanding the clonal dynamics for both cases is of importance. Here, we have focused on the accumulation of non-stem cell-driven mutations in hierarchically organized tissues. We arrived at closed solutions for the clonal waves travelling through the hierarchy. From this, the reproductive capacity of cells can be deduced. This allows us to predict the expected risk of acquiring any number of mutations from one single cell, given the proliferation properties of this cell. We derived equations that allow the quantitative classification of multiple mutations in hierarchically organized structures, highlighting the strong suppression of clones carrying multiple mutations by this architecture. Although we neglected clonal competition over limited resources, such as cytokines or nutrients, we expect the model to capture general patterns of clonal expansions in hierarchically organized tissues and to serve as a null model. Moreover, those cancer cells that divide independently of proliferation signals (for example, owing to mutated tyrosine kinases) escape such a competition.

Another important question emerged more recently with the accessibility of whole genome sequencing technology. These techniques revealed very complex and diverse mutation landscapes for many different cancers, with the classification of driver and passenger mutations as the final goal. Knowing the exact driver mutations might help our understanding of the properties of specific cancer cells, allowing the development of effective treatment strategies. A promising example is the design of molecularly targeted agents such as the various tyrosine kinase inhibitors (imatinib, nilotinib, etc.) to treat patients with chronic myeloid leukaemia. These molecules specifically bind to kinase domains encoded by the BCR-ABL oncogene and strongly suppress the proliferative capacity of these cells [58–60]. Our work can contribute to this question by predicting the average number of distinct neutral (passenger) mutations acquired from a single cell at any stage of the hierarchy. This approach directly corresponds to the method of a neutral marker. The genetically marked cell represents the founder cell of the clonal population and one can follow the offspring of this founder cell throughout the hierarchy. This enables the prediction of the size and the variability of the clonal population. For example, in normal haematopoiesis, we expect cells to have a differentiation probability of *ɛ* = 0.85, leading to approximately 30 distinct single mutations (subclones) in adult cells acquired from this single cell. If this founder cell acquired a mutation that changed the differentiation probability to *ɛ* = 0.75 by chance, the expected number of distinct single mutations increases to approximately 28 000.

We have shown that even a slight change in the self-renewal probability of progenitor cells can lead to substantial differences in the number of passenger mutations observed in ALL. This probably holds true for other malignancies. In acute promyelocytic leukaemia, apart from the t(15;17) that is a critical event in the origin of this disease (akin to the ETV6–RUNX1 discussed earlier for ALL), approximately 440 non-synonymous single nucleotide mutations were found, which were unique to the tumour clone [21]. Interestingly, it is highly likely that the cell of origin of acute promyelocytic leukaemia is downstream (a progenitor cell) of the cell of origin of ETV6–RUNX1-driven ALL and this may, in part, explain the less diverse mutational landscape reported in acute promyelocytic leukaemia compared with ALL and would fit well with our model. Of course, any genomic instability will further increase the repertoire of passenger mutations that is observed in any given tumour.

We also note that the effect of a specific mutation on a cell needs not be large for the effect to spread throughout the tumour. Tissue architecture and dynamics, such as in haematopoiesis, serve as a deterrent against the accumulation of mutations, in particular multiple mutations occurring in one cell. Once a driver mutation appears, if this changes either the self-renewal of the cell or the mutation rate, then the appearance of many passenger mutations becomes inevitable in such an architecture because of the amplification of cells that occurs. Thus, a minor change in the differentiation probabilities that might be difficult to detect *in vivo* drastically changes the expected number of passenger mutations that would be observed. Concomitantly, this will increase the risk of acquiring mutations in ‘driver genes’ and so lead to malignancy. However, if the initiation of disease requires several co-occurring mutations, a hierarchical tissue structure is a powerful mechanism of tumour suppression.
